# Both JNK1 and JNK2 Are Indispensable for Sensitized Extracellular Matrix Mineralization in IKKβ-Deficient Osteoblasts

**DOI:** 10.3389/fendo.2020.00013

**Published:** 2020-02-12

**Authors:** Qianyun Hao, Zhuangzhuang Liu, Liaoxun Lu, Lichen Zhang, Li Zuo

**Affiliations:** ^1^Department of Nephrology, Peking University People's Hospital, Beijing, China; ^2^Laboratory of Mouse Genetics, Institute of Psychiatry and Neuroscience, Xinxiang Medical University, Xinxiang, China; ^3^Laboratory of Genetic Regulators in the Immune System, Henan Collaborative Innovation Center of Molecular Diagnosis and Laboratory Medicine, Xinxiang Medical University, Xinxiang, China

**Keywords:** osteoblasts, IKKβ, NF-κB, JNK1, JNK2, CRISPR/Cas9

## Abstract

Extracellular matrix mineralization is critical for osteogenesis, and its dysregulation could result in osteoporosis and vascular calcification. IKK/NF-κB activation inhibits differentiation of osteoblasts, and reduces extracellular matrix mineralization, however the underlying mechanisms are poorly understood. In this study, we used CRISPR/Cas9 system to permanently inactivate IKKβ in preosteoblast cells and confirmed that such cells displayed dramatic increase in extracellular matrix mineralization associated with JNK phosphorylation. Such observation was also found in our study using IKKβ-deficient primary murine osteoblasts. Interestingly, we found that in *Ikbkb*^−/−^ *Mapk8*^−/−^ or *Ikbkb*^−/−^ *Mapk9*^−/−^ double knockout cells, the enhanced mineralization caused by IKKβ deficiency was completely abolished, and deletion of either *Mapk8* or *Mapk9* was sufficient to dampen c-Jun phosphorylation. In further experiments, we discovered that absence of JNK1 or JNK2 on IKKβ-deficient background resulted in highly conserved transcriptomic alteration in response to osteogenic induction. Therefore, identification of the indispensable roles of JNK1 and JNK2 in activating c-Jun and promoting osteoblast differentiation on IKKβ-deficient background provided novel insights into restoring homeostasis in extracellular matrix mineralization.

## Introduction

Bone formation requiring extracellular matrix mineralization is an active, highly regulated process primarily mediated by osteoblasts (1). Dysfunction of mineralization could ultimately lead to adverse clinical outcomes such as skeletal fragility and vascular calcification (2, 3). Compelling evidences show that NF-κB regulates bone metabolism for its roles in facilitating osteoclasts differentiation, although mechanisms underlying NF-κB in osteoblast differentiation and matrix mineralization are little known (4–7). IKKβ is the major catalytic subunit of the IKK complex and is required for the activation of NF-κB by inflammatory mediators in the canonical or classical activation pathway (8). It is known that inhibition of IKK/NF-κB promotes mineralization in osteoblasts (9), but the underlying mechanisms are poorly understood. In addition, there remain contradictory reports on some agents that can induce osteoblast differentiation and mineralization through activating NF-κB pathway (10, 11). The IKK/NF-κB pathway which has essential and complex roles in matrix mineralization requires further mechanistic studies. Furthermore, previous studies showed that phosphorylation of JNK was found altered in osteoblasts in the presence of IKK/NF-κB inhibition (9). However, it remains unknown whether JNK activation itself necessitates increased matrix mineralization in osteoblasts with IKK/NF-κB inhibition. More importantly, JNK consists of three members, JNK1 and JNK2 express in ubiquitous tissues and cells, while JNK3 is limited in brain, testis and heart (12, 13), but the roles of JNK, particularly those of its different members in osteoblast differentiation and mineralization remain weakly studied. In addition, previous studies presented conflicting reports regarding the roles of JNK1 and JNK2 in osteogenesis. One study reported that differentiation of osteoblasts and matrix mineralization were significantly promoted by over expression of JNK2 but not affected by over expression of JNK1 (14), while other studies emphasized the important role of JNK1 (15, 16). Therefore, it is highly necessary to perform mechanism studies by means of genetic models to unambiguously determine the roles of JNK1 and JNK2 in mineralization. MC3T3–E1 cells can be induced by β-glycerophosphate (β-GP) and ascorbic acid (AA) and result in matrix mineralization (14). In this study, we performed genetic inactivation of IKKβ in preosteoblast cells MC3T3–E1 and primary osteoblasts harvested from neonatal mice. By CRISPR/Cas9 genome editing tool, we isolated stable clones of mutant MC3T3-E1 cells completely deprived of IKKβ protein, which allowed us to establish a cellular model extremely sensitive to induced matrix mineralization. We discovered that IKKβ deletion in MC3T3-E1 and murine primary osteoblasts significantly increased JNK phosphorylation. Furthermore, we deleted JNK1 or JNK2 under the background of IKKβ deficiency to disambiguate the functions and mechanisms of these molecules. It is important to note that MC3T3–E1 cells display relatively mild matrix mineralization upon osteogenic induction medium treatment, while IKKβ deficiency enables dramatic increase of their capacity for differentiation and mineralization. In this sensitized model, we discovered that both JNK1 and JNK2 were indispensably required for osteogenesis and knockout of either JNK1 or JNK2 completely abolished IKKβ deficiency-enhanced osteoblast differentiation and mineralization. We also found that c-Jun activation required both JNK1 and JNK2 in IKKβ-deficient MC3T3–E1 cells. Strikingly, our transcriptomic analyses revealed that JNK1 and JNK2 contributed very similarly to the response of IKKβ-deficient MC3T3–E1 cells to osteogenic induction. Therefore, our study using genetic models on the IKKβ-deficient background confirmed the decisive roles of both JNK1 and JNK2 in mineralization.

## Materials and Methods

### Plasmid Construction

N20NGG motifs in the *Ikbkb, Mapk8* and *Mapk9* (genes encoding IKKβ, JNK1, and JNK2) loci were screened, respectively, and candidates sgRNAs that match the criteria for U6 promoter transcription were selected for CRISPR/Cas9 knockout (17). Three sgRNAs were designed for each gene knockout and sequences are listed in Supplementary Table 1. Oligonucleotides were annealed and cloned, respectively, into pX458 or its derivatives (pX458-DsRed2 and pX458-ECFP) as described previously (18).

### Cell Line Culture and Transfection

The mouse preosteoblast cell line MC3T3–E1 was obtained from the Cell Resource Center, IBMS, CAMS/PUMS (Beijing, China). Cells were cultured in basic medium, α-MEM (Gibco BRL, Grand Island, NY, USA) supplemented with 10% FBS (Hyclone, Logan, UT, USA), 100 U/ml penicillin, 100 μg/ml streptomycin (Gibco BRL, Grand Island, NY, USA), and were maintained in a humidified, 5% CO_2_ atmosphere at 37°C according to the recommended procedures by American Type Culture Collection (ATCC). MC3T3–E1 cells were seeded in 12-well plates 24 h before transfection with 70% confluency. Cells in each well were transfected with 2 μg of *Ikbkb* targeting plasmids (pX458-ECFP-m-*Ikbkb*-KO-1, pX458-ECFP-m-*Ikbkb*-KO-2 and pX458-DsRed2-m-*Ikbkb*-KO-3) using Lipofectamine® 3000 Transfection Kit (Invitrogen, Carlsbad, CA, USA) according to the manufacturer's protocol.

### Isolation of Gene-Knockout Cells

MC3T3–E1 cells were collected and subjected to fluorescence-activated cell sorting (FACS) assay at 48 h post-transfection. We used non-transfected MC3T3–E1 cells as negative control, among the transfected cells, DsRed2/ECFP double-positive population was regarded as having been transfected with the CRISPR/Cas9 system successfully. Expanded clones were harvested for genomic DNA analysis when close to confluence. To generate IKKβ-deficient bulk-sorted cells, we transfected MC3T3–E1 cells with *Ikbkb* targeting plasmids, and we sorted 10,000 cells in one well of 24-well plate to expand. One of the IKKβ-deficient clones was further transfected with *Mapk8* or *Mapk9* targeting plasmids (listed in Supplementary Table 1) respectively, and were sorted by FACS as *Ikbkb*^−/−^ *Mapk8*^−/−^ or *Ikbkb*^−/−^ *Mapk9*^−/−^ clones.

To genotype the mutant clones, we used agarose gel electrophoresis analysis of PCR products. Such experiments were performed to screen for monoclonal mutant cells as each locus in our study was targeted by two or more independent sgRNAs which could result in large DNA fragment deletions, as described in our previous study (18). PCR products were cloned into the pBLUE-T vector using the pBLUE T Fast Cloning Kit (Zoman Biotechnology, China) and subjected to Sanger sequencing.

### Osteoblast Differentiation and Mineralization Assay

To induce osteoblast differentiation and mineralization, we seeded MC3T3–E1 cells in 6-well plates, osteogenic medium (α-MEM containing 10% FBS, 100 U/ml penicillin, 100 μg/ml streptomycin, 10 mM β-GP and 50 μg/ml AA) was used to culture the cells 24 h after seeded (designated as day 0). AA and β-GP were purchased from Sorlabio (Beijing, China) and Sigma-Aldrich (St. Louis, MO, USA), respectively. The medium was changed every 2 days for 8, 12, 16, and 20 days of induction. In addition, we induced osteoblast differentiation and mineralization by BMP-2 (100 ng/ml, R&D Systems, MN, USA) with the addition of 50 μg/mL AA and 5 mM β-GP in the culture medium for 12, 16 days.

### ALP Assay and Alizarin Red S Staining Assay

Alkaline phosphatase (ALP) assay was performed at day 8 of osteogenic induction. We fixed cells with 4% paraformaldehyde, and stained with an ALP staining kit (Beyotime, China) according to the manufacturer's protocol. Cells were stained for 15 min at room temperature, and the staining was stopped by washing with distilled water for three times.

The matrix mineralization of cells was determined by Alizarin Red S staining at day 8, 12, 16, 20 of osteogenic induction. The cultured cells were fixed with 4% paraformaldehyde, and stained with 1% Alizarin Red S (Sigma-Aldrich, St. Louis, MO, USA), pH 4.2, for 10 min at room temperature. Then, cells were washed with distilled water for three times to eliminate non-specific staining. To quantify extracellular matrix mineralization, the bound stain was de-stained with 10% (w/v) cetylpyridinium chloride in water at room temperature for 30 min, followed by diluting with the same volume of 10% (w/v) cetylpyridinium chloride, and then quantified by measuring absorbance at 560 nm.

### Intracellular Labeling Phosphorylated Proteins and FACS Analysis

The phosphorylation of JNK and c-Jun in MC3T3–E1 wildtype (WT) and genomic mutation cells in response to stimulation of β-GP and AA were determined according to a modified eBioscience protocol for staining intracellular antigens. Briefly, at the beginning of the experiment, basic medium (α-MEM containing 10% FBS) was changed to osteogenic medium (α-MEM containing 10% FBS, 10 mM β-GP, and 50 μg/ml AA) or basic medium containing BMP-2 (α-MEM containing 10% FBS, 100 ng/ml BMP-2). After exposure to different stimulation time points, the stimulation was stopped by adding Lyse/Fix Buffer BD Phosflow™ (BD Biosciences, San Diego, CA, USA) or eBioscience™ Intracellular Fixation Buffer (Invitrogen, San Diego, CA, USA) to fix the cells according to the manufacturer's protocol. After washing with FACS buffer (1 × DPBS, 1% bovine serum, 2 mM EDTA), cells were permeabilized with 90% methylalcohol (Sigma-Aldrich, St. Louis, MO, USA). And then, cells were stained with specific primary antibodies for 90 min at 4°C. Finally, cells were exposed to the secondary antibody Allophycocyanin-conjugated goat anti-rabbit (1:1,000, Life technology, Eugene, OR, USA) for 30 min after washing with FACS buffer, and then, mean fluorescence intensity (MFI) was measured by BD FACSCanto (BD Biosciences). The data was analyzed using Flowjo version 10.1 software (TreeStar Inc., Ashland, OR, USA). The following primary antibodies were used: phospho-SAPK/JNK (1:200, Cell Signaling Technology, #9251), phospho-c-Jun (1:200, Cell Signaling Technology, #9261).

### Primary Calvarial Osteoblasts Culture and Transfection

Primary calvarial osteoblasts were isolated from 48-h-old newborn C57BL/6 mice. C57BL/6 mice were purchased from Beijing Vital River Laboratory Animal Technology Co., Ltd., and were housed under pathogen-free conditions. The animal procedure was conducted according to guidelines approved by the committee on animal care at Xinxiang Medical University. After removal of calvarias, the tissues were digested with Liberase DH Research Grade (Roche, Mannheim, Germany) for 15 min. Then, the supernatant was discarded and the remaining bone tissue was continually digested for another 15 min. The supernatant was collected and maintained in α-MEM supplemented with 15% fetal bovine serum. To induce osteoblast differentiation, the medium was changed to osteogenic medium after the cells were sub-cultured. ALP and Alizarin Red S staining assay were performed at day 8 and day 21 of induction, respectively.

To compare the different level of phosphorylated JNK between the wildtype primary osteoblasts and the IKKβ-deficient ones, we cloned *Ikbkb* targeting sgRNA1, 2, 3, respectively, into plasmid pX458. Primary osteoblasts with the second passage were used for this experiment. The transfection was performed 24 h after the primary osteoblasts were sub-cultured and seeded in 6-well plates with 90% confluence. Primary cells were transfected with 5 μg of pX458 empty backbone plasmid or *Ikbkb* targeting plasmids using Lipofectamine® 3000 Transfection Kit (Invitrogen, Carlsbad, CA, USA). At 48 h post-transfection, the primary osteoblasts transfected with pX458 empty backbone plasmid or *Ikbkb* targeting plasmids were stimulated with β-GP and AA for 30 min, and were fixed by Lyse/Fix Buffer BD Phosflow™, and then subjected to intracellular labeling phospho-JNK and FACS analysis.

### Western Blot Analysis

After stimulation, cells were lysed with RIPA lysis buffer (Beyotime, China) with freshly added protease and phosphatase inhibitor cocktails. After incubation on ice for 30 min with frequent vortex, the resulting cell lysates were collected and centrifuged (13,000 × g, 15 min, 4°C), and the proteins were boiled in loading buffer (Beyotime, China) at 100°C for 10 min. Equal amounts of proteins from each sample were separated on 8% SDS-PAGE gel and then electrophoretically transferred onto PVDF membranes (Millipore, Bedford, MA, USA). The membrane was blocked with 5% non-fat milk in Tris-buffered saline containing 0.1% T-ween 20 (TBST) for 60 min at room temperature, and cultured with specific primary antibody in TBST with 5% bovine serum albumin (BSA, Sigma-Aldrich, St. Louis, MO, USA) overnight at 4°C. After the incubation, the membrane was washed 3 times with TBST and exposed to the appropriate HRP-conjugated secondary antibody (1:3,000, Cell Signaling Technology) in TBST with 5% non-fat milk for 60 min at room temperature. After subsequent washing in TBST, blots were visualized using Immobilon® Western Chemiluminescent HRP Substrate kit (Millipore, Bedford, MA, USA). All experiments were performed at least in triplicate. The primary antibodies were used to detect molecules: phospho-SAPK/JNK (1:1,000, Cell Signaling Technology, #9251), SAPK/JNK (1:1,000, Cell Signaling Technology, #9252), JNK1 (1:1,000, Cell Signaling Technology, #3708), JNK2 (1:1,000, Cell Signaling Technology, #9258), phospho-Smad1/5 (1:1,000, Cell Signaling Technology, #9516P), Smad1 (1:1,000, Cell Signaling Technology, #9743P), phospho-c-Jun (1:1,000, Cell Signaling Technology, #9261), phospho-NF-κB p65 (1:1,000, Cell Signaling Technology, #3033), NF-κB p65 (1:1,000, Cell Signaling Technology, #8242), IKKβ (1:1,000, Cell Signaling Technology, #8943), β-Actin (1:1,000, Beyotime, AA128).

### Immunofluorescence

Cells plated on glass covers 24 h before the experiment. Basic medium was changed to osteogenic medium, and after exposure to specific stimuli time, the cells were fixed with Lyse/Fix Buffer BD Phosflow™ (BD Biosciences, San Diego, CA, USA). After washing with PBS for 3 times, the cells were permeabilized with 0.1% TritonX-100 and blocked with 3% BSA for 60 min at room temperature. Afterwards, the cells stained with primary antibodies against phospho-JNK (1:100, Cell Signaling Technology, #9251) or phospho-c-Jun (1:100, Cell Signaling Technology, #9261) in 1% BSA over night at 4°C. Samples were then washed with PBS and incubated for 1 h at 4°C with Alexa Fluor 488-conjugated goat anti-rabbit antibody (1:500, Invitrogen, Carlsbad, CA, USA) in 1% BSA. Nuclei were stained with DAPI. Leica TCS SP8 STED 3X confocal microscope was used for imaging the cells, and the images were processed and overlaid with Leica Application Suite X.

### RNA Extraction and Real-Time PCR

Total RNA was extracted from cultured cells using the RNeasy Plus kit (Qiagen, Duesseldorf, Germany), and the quantity and integrity of RNA were evaluated using NanoDrop One (Thermo Fisher Scientific). A total of 2 μg RNA was reversed-transcribed into cDNA using the RevertAid First Strand cDNA Synthesis Kit (Thermo Fisher Scientific, Vilnius, Lithuania), and the Real-time PCR reaction was performed using TB Green™ Premix Ex Taq™ (TaKaRa, Japan) and specific primers (Supplementary Table 2) with the StepOnePlus Real-time PCR system (Applied Biosystems, Life technologies). Transcript level of each mRNA was normalized by comparison with mRNA of *Gapdh*, and was calculated using the 2^−ΔΔCT^ method.

### RNA-seq Analysis

After *Ikbkb*^−/−^ knockout cells, *Ikbkb*^−/−^ *Mapk8*^−/−^, and *Ikbkb*^−/−^ *Mapk9*^−/−^ double knockout cells were treated with basic medium and osteogenic medium, respectively, for 4 days (*n* = 3 per group), total RNAs were isolated by RNeasy Plus kit (Qiagen, Duesseldorf, Germany), subjected to RNA sequencing analysis (BGI, Ltd., Shenzhen, China) on the BGISEQ-500 platform and analyzed deferentially expressed genes (DEGs). Gene expression was calculated using the FPKM method (19), DEGSeq method was utilized to identify differentially expressed genes (20), |log2 (FC)| >1 and adjusted *P* < 0.001 were determined as the significant change threshold. The significant GO terms and important pathways based on the latest KEGG database were assessed based on the threshold of *P* < 0.05. Data were deposited in the NCBI Sequence Read Archive (SRA) database (www.ncbi.nlm.nih.gov/sra, accession number SRP219248).

### Data Analysis

Data are presented as mean ± SEM. All statistical methods were analyzed by two-tailed test, with *P* < 0.05 considered statistically significance. Comparisons between two groups were performed by the *t*-test, the statistical significance of differences with more than two groups was assessed via one-way ANOVA, and multiple comparisons between the different groups were made using the Bonferroni test.

## Results

### Genetic Ablation of IKKβ in MC3T3-E1 Preosteoblast Cells

**Figure 1:**
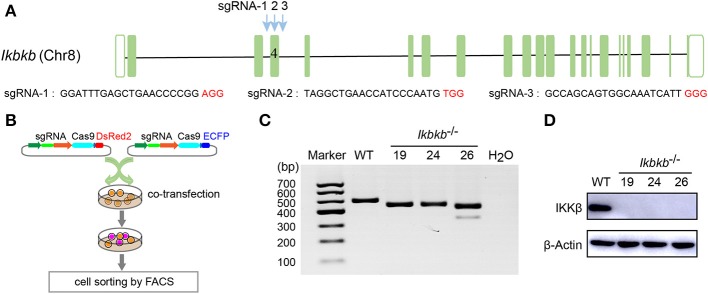
Deletion of IKKβ in MC3T3–E1 cells. **(A)** Schematic diagram of the sgRNAs designed to inactivate mouse IKKβ, which were placed inside *Ikbkb* exon 4. Three PAM motifs were shown in red color. **(B)** Workflow for generation of mutant cells in MC3T3–E1. Generally, two or more independent sgRNAs were designed and expressed in different vectors which express ECFP or DsRed2 as reporters. 48 h following transfection, cells simultaneously expressing two reporter proteins were subjected to cell sorting by FACS. Genotyping of the mutant clones was done by running electrophoresis with the PCR amplicons on agarose gel. **(C)** *Ikbkb*^−/−^ clone 19, 24, and 26 were selected by PCR and agarose electrophoresis. CRISPR/ Cas9-mediated genomic editing resulted in the PCR products of *Ikbkb*^−/−^ clone 19, 24, and 26 around 40 and 150 bp smaller than that of the wildtype (WT) control. H_2_O was used as negative control. **(D)** Representative Western blot for total IKKβ protein levels of MC3T3–E1 WT cells and CRISPR/Cas9-mediated *Ikbkb*^−/−^ cells.

MC3T3–E1 cells are derived from C57BL/6 newborn mouse calvaria, and have the capacity to differentiate and mineralize, which provides a cellular model for mineralization study (14). IKKβ is the major subunit of the IKK complex and is required for the activation of NF-κB (8). It has been reported that inhibition of IKK/NF-κB in osteoblasts promotes mineralization (9). Therefore, it is desirable to obtain genetic model deficient in IKKβ with sensitized mineralization phenotype for in-depth mechanism studies. First, we sought to genetically delete *Ikbkb* gene and permanently remove functional IKKβ protein to inactivate IKK/NF-κB pathway in MC3T3-E1 preosteoblast cells by CRISPR/Cas9. As described in our previous study (18), multiple sgRNAs were designed to target a critical exon, which could generate large DNA fragment deletions. In this study, the transcript ENSMUST00000033939.12 archived by Ensembl which encodes a protein of 757 amino acids was selected, and the conserved exon 4 was targeted by three independent guide RNAs (Figure 1A). It is important to note that we used transient expression of the CRISPR/Cas9 system after transfection with plasmids engineered with dual fluorescent reporters, which could avoid drug selection and long-term exposure of transfected cells to DNA cleavage (Figure 1B). Single cell sorting was performed and several desirable clones harboring bi-allelic DNA fragment deletions were isolated after clonal expansion (Figure 1C and Supplementary Figure 1A). We performed DNA sequencing of the selected clones and validated that each had DNA deletion at targeted loci, as shown in Supplementary Figure 1B. *Ikbkb*^−/−^ clone 19 had a 42 & 4-bp deletion in exon 4, and another clone named *Ikbkb*^−/−^ clone 24 had mutations at two alleles, one with 42-bp deletion and the other allele with 42-bp plus 3-bp deletion. *Ikbkb*^−/−^ clone 26 had two alleles, one carried 42 & 3-bp deletion, while the other allele had 153-bp fragment deletion. The DNA deletion resulted in complete absence of protein as detected by Western blot (Figure 1D). The data showed that three independent mutant clones of MC3T3-E1 cells completely deprived of IKKβ protein were obtained by CRISPR/Cas9 genome editing tools.

### IKKβ Deficiency Extremely Enhances Osteoblast Differentiation and Extracellular Matrix Mineralization in MC3T3-E1 Cells

**Figure 2:**
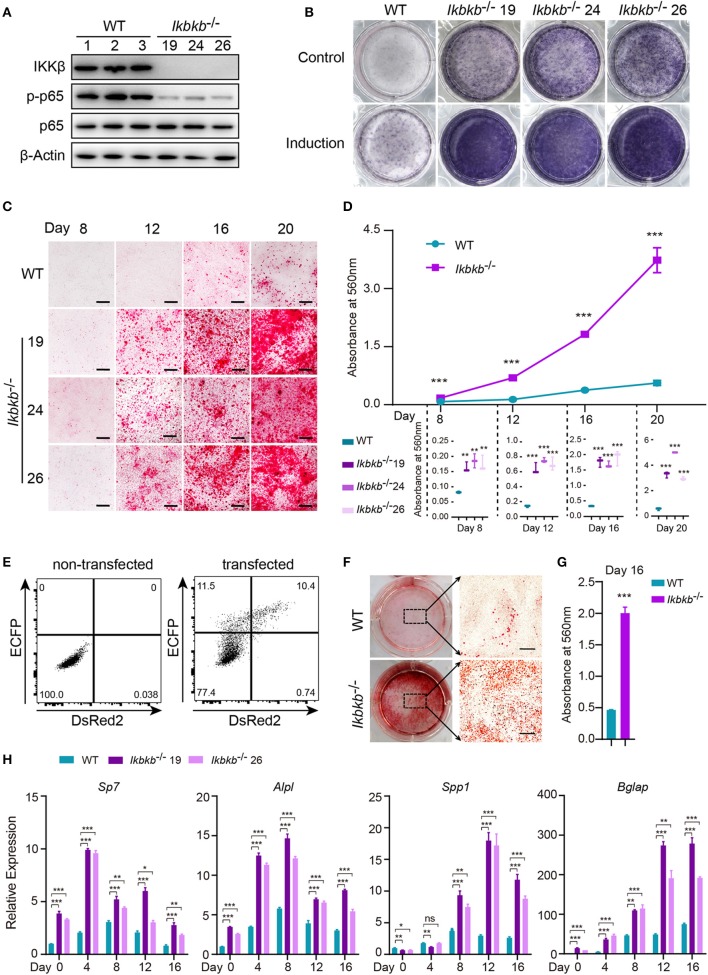
Enhanced osteogenic differentiation and matrix mineralization in IKKβ-deficient MC3T3–E1 cells. **(A)** Phosphorylation of p65 was examined by Western blot analysis in steady state. The phosphorylation of p65 at Ser536, an important site of IKKβ action was significantly inhibited. **(B)** The wildtype (WT) control and *Ikbkb*^−/−^ clone 19, 24, 26 were cultured in the control medium or osteogenic medium for 8 days. ALP activity was analyzed using ALP staining. **(C)** Microscopy images of Alizarin Red S staining of WT control and *Ikbkb*^−/−^ cells induced by osteogenic medium. **(B,C)** Results were representatives of three independent experiments. **(D)** Mineralization described in **(C)** was quantified by de-staining Alizarin Red S with 10% cetylpyridinium chloride and the absorbance was measured at 560 nm (*n* = 3), the level of mineralization in *Ikbkb*^−/−^ cells was compared with WT cells. Statistics by one-way ANOVA. **(E)** FACS plots showing non-transfected control and cells transfected with *Ikbkb* targeting plasmids. We sorted 10,000 DsRed2/ECFP double-positive cells as *Ikbkb*^−/−^ cells bulk, and subjected to phenotype experiment. Alizarin Red S staining **(F)** and quantification **(G)** of *Ikbkb*^−/−^ cells bulk induced by osteogenic medium for 16 days compared with WT cells (*n* = 3). Statistics by two-tailed *t*-test. **(H)** The deletion of IKKβ increased mRNA levels of osteoblast differentiation marker genes as determined by real-time polymerase chain reaction (*n* = 3), compared between WT and IKKβ-deficient cells. Statistics by one-way ANOVA. Scale bars: 500μm. Error bars represent ± SEM. *P < 0.05, **P < 0.01, ***P < 0.001, ns = not significant.

Before characterizing the phenotype of mutant cells deprived of IKKβ protein, we first performed Western blot analysis to examine phosphorylation of p65 which is crucially required for NF-κB activation (21). As expected, we found that p65 phosphorylation in all mutant clones were dramatically diminished, with p65 total protein and β-Actin as loading controls (Figure 2A). To assess osteoblast differentiation and mineralization capacity of the genetic mutant clones, ALP staining and Alizarin Red S staining were performed by culturing the cells in basic medium as control and osteogenic induction medium which was supplemented with AA and β-GP for indicated days. As ALP is one of the most important markers of osteoblast differentiation (22), our results showed that IKKβ deficiency obviously increased ALP activity (Figure 2B). In addition, deletion of IKKβ led to significantly higher level of extracellular matrix mineralization, as assessed by Alizarin Red S staining. The most obvious difference appears at day 20 of differentiation, the absorbance at 560 nm of three independent IKKβ-deficient clones was 6.7-fold higher than that in WT cells (Figures 2C,D; Images of 6-well plates presented in Supplementary Figure 2A). To solidify the results observed in three mutant clones, we further validated in cells undergone CRISPR/Cas9 plasmids transfection and bulk-sorting by FACS which gave rise to mutant cells including maximal number of deleterious alleles (18). We transfected MC3T3–E1 cells using the CRISPR/Cas9 plasmids with dual fluorescent reporters as illustrated in Figure 2E, involving the three sgRNAs as described above. The sgRNAs targeting *Ikbkb* were found to be highly efficient and the bulk-sorted cells contained a large variety of mutant alleles analyzed with capillary array electrophoresis following fluorescent PCR, and WT allele was hardly detected in the sorted cells (Supplementary Figures 2B,C). Strikingly, the bulk-sorted cells like three individual mutant clones had dramatically increased matrix mineralization in osteogenic medium, as assessed by Alizarin Red S staining (Figures 2F,G). In further experiments, we analyzed the osteoblast differentiation markers including mRNAs of *Sp7* (encoding Osterix, Osx), *Alpl* (encoding alkaline phosphatase, ALP), *Spp1* (encoding Osteopontin, OPN), *Bglap* (encoding Osteocalcin, OC), *Runx2* (encoding Runt-related transcription factor 2, Runx2) following 4, 8, 12, 16 days of osteogenic induction. We found that *Sp7, Alpl*, and *Bglap* reflecting osteoblast differentiation in IKKβ-deficient cells increased by 3.6–9.5-folds compared with those in WT cells at mRNA levels when analyzed at day 4 (Figure 2H). The important osteoblast marker OPN (encoded by *Spp1*) of WT and IKKβ-deficient cells had no significant difference at mRNA level after 4 days of induction, but it was ~3 times and ~6 times more abundant in IKKβ-deficient cells than in WT cells when analyzed at day 8 and day 12, while the other markers such as *Sp7* and *Alpl* had most pronounced increase in expression in IKKβ-deficient cells at day 4, and *Bglap* had most pronounced increase at day 12 (Figure 2H). *Runx2* expression had most difference at day 4 but the increase was mild (Supplementary Figure 2D). We also analyzed WT cell expression of differentiation markers in steady state or in induction medium, and parallel analyses were performed with IKKβ-deficient cells. The pattern for marker expression during osteogenic induction was similar but the increase was apparently more obvious in IKKβ-deficient cells (Supplementary Figure 2F). Taken together, our experiments using individual clones and bulk-sorted cells validated in an unambiguous manner that loss of IKKβ rendered MC3T3–E1 cells highly sensitive to osteogenic induction. Such IKKβ-deficient MC3T3–E1 cells provided a special model for mineralization investigation.

### IKKβ Deficiency Results in JNK Activation Both in Primary Osteoblasts and MC3T3-E1 Cells

**Figure 3:**
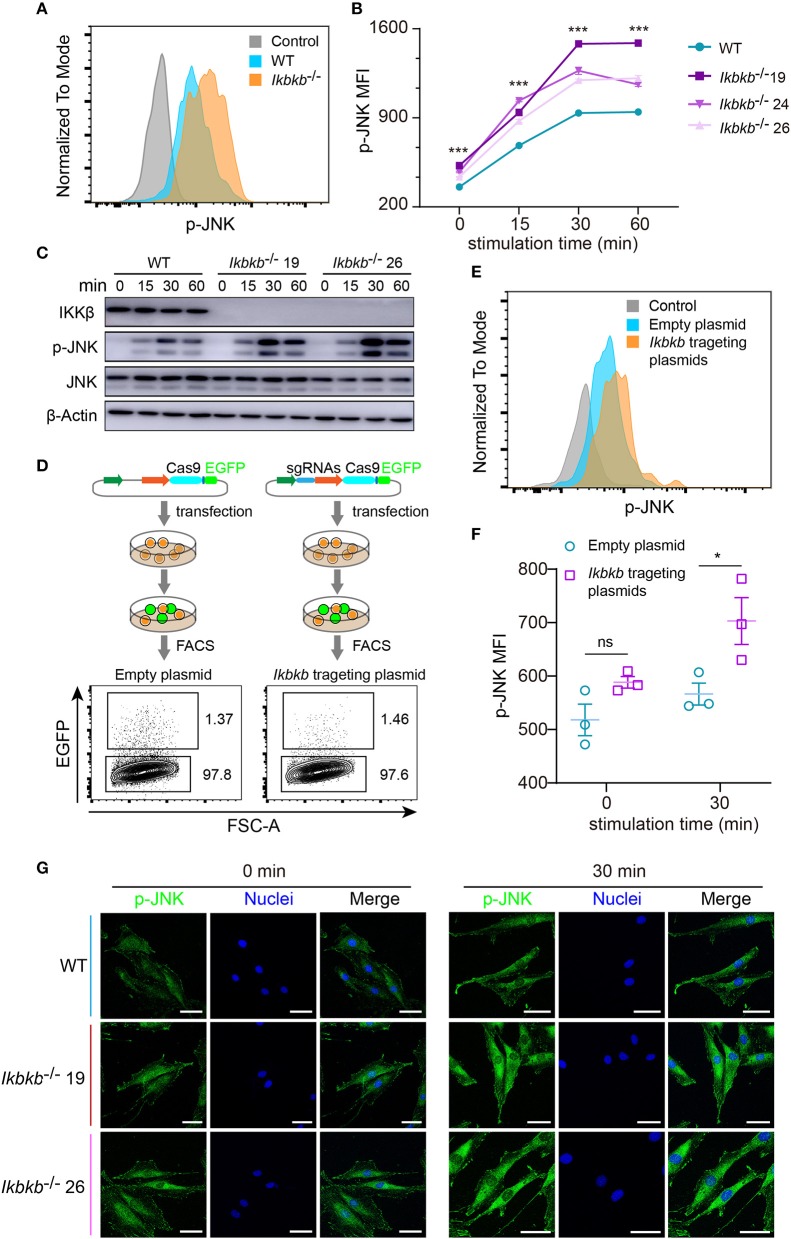
Deletion of IKKβ increases phosphorylation of JNK in both MC3T3–E1 cells and primary calvarial osteoblasts. **(A,B)** Flow cytometry analysis of phospho-JNK in MC3T3–E1 wildtype (WT) cells and IKKβ-deficient cells after β-GP and AA stimulation. **(A)** Representative data of phosphorylation of JNK at 30 min after β-GP and AA stimulation. Fluorescence intensity for phospho-JNK was compared using histogram overlay between fluorescence minus one (FMO) control (gray), WT cells (Blue), and IKKβ-deficient cells (Orange). **(B)** Mean fluorescence intensity (MFI) of phospho-JNK at 0, 15, 30, and 60 min after β-GP and AA treatment, compared between WT and IKKβ-deficient cells (*n* = 6). Statistics by one-way ANOVA. **(C)** Western blot analysis for phospho-JNK in WT and IKKβ-deficient MC3T3–E1 cells after stimulating by β-GP and AA for indicated time points. Results were representatives of three independent experiments. **(D)** FACS plots showing primary osteoblasts transfected with pX458 empty backbone plasmid (left panel) as control and pX458-*Ikbkb*-targeting plasmids (right panel). The plasmids could express EGFP reporter. By gating on the EGFP positive primary osteoblasts, we distinguished the two groups of cells which were successfully transfected with empty backbone plasmid or IKKβ targeting plasmids, and compared their MFI of phospho-JNK at 0, 30 min after β-GP and AA stimulation **(E,F)**. **(E)** Showing representative data at 30 min after β-GP and AA treatment. **(F)** Differences of phospho-JNK MFI between *Ikbkb* targeting primary osteoblasts and backbone plasmid transfected cells, with or without β-GP and AA treatment (*n* = 3). Statistics by two-tailed *t*-test. **(G)** Representative immunofluorescence images of phospho-JNK (green) and nuclei (blue) staining in WT and IKKβ-deficient MC3T3–E1 cells at 0, 30 min after the stimulation by β-GP and AA. Scale bars: 50 μm. Error bars represent ± SEM. *P < 0.05, ***P < 0.001, ns = not significant.

JNK pathway has crosstalk with IKK/NF-κB and can be up-regulated by inhibition of IKK/NF-κB (9, 23). Function of JNK pathway is complicate and varies in different cell types, which induces apoptosis in many cell types but stimulates cell proliferation in epidermal cells (23). More importantly, previous studies of the function of JNK in osteoblasts remain controversial (16, 24), therefore it is highly necessary to perform analyses in a stable genetic model, desirably on the IKKβ-deficient sensitized background. Since MC3T3–E1 cells and primary osteoblasts can be induced toward differentiation and matrix mineralization by osteogenic medium (basic medium supplemented with β-GP and AA), we explored intracellular signal in response to β-GP and AA stimulation. Using flow cytometry and intracellular labeling of phosphorylated JNK, we found that MC3T3-E1 cells deprived of IKKβ displayed significant increase in JNK phosphorylation following β-GP and AA stimulation at various time points (Figures 3A,B). Importantly, by immunoblotting we found that in mutant cells which were absent in IKKβ proteins, phosphorylation of JNK proteins were potently induced at 15, 30, and 60 min after osteogenic medium treatment, while the WT control cells had obviously less intensity in phosphorylated JNK bands (Figure 3C). In further experiments, we used IKKβ-deficient primary osteoblasts harvested from neonatal mice with reference to a modified protocol (25). Before subject to genome editing, the primary osteoblasts were validated for their capacity to differentiate and mineralize *in vitro* (Supplementary Figure 3). Three independent sgRNAs targeting *Ikbkb* as illustrated in Figure 1A were cloned to pX458 plasmid which also encoded EGFP reporter. By gating on the EGFP expressing murine primary osteoblasts which were successfully transfected either empty backbone plasmid or *Ikbkb* targeting plasmids (Figure 3D), we compared JNK phosphorylation upon osteogenic medium treatment. In such primary cells, we found that *Ikbkb* targeting by CRISPR/Cas9 resulted in significant increase of JNK phosphorylation as measured by MFI following 30 min of stimulation by β-GP and AA when compared to empty plasmid transfected cells (Figures 3E,F). With quantification of JNK phosphorylation on single cell basis by using FACS analysis, we performed confocal microscopic study of the IKKβ-deficient cells to assess localization and abundance of phosphorylated JNK in MC3T3-E1 cells. We observed consistent results that phosphorylated JNK was more abundant in IKKβ-deficient cells with distribution mainly in cytosol but also visible in nucleus following osteogenic medium treatment (Figure 3G). Therefore, our results from both primary osteoblasts and MC3T3-E1 cells showed that IKKβ deficiency resulted in activation of JNK, suggesting that hyperactivity of JNK could be involved in enhanced osteoblast differentiation and mineralization.

### Hyperactive c-Jun During Osteoblast Differentiation in IKKβ-Deficient Cells

**Figure 4:**
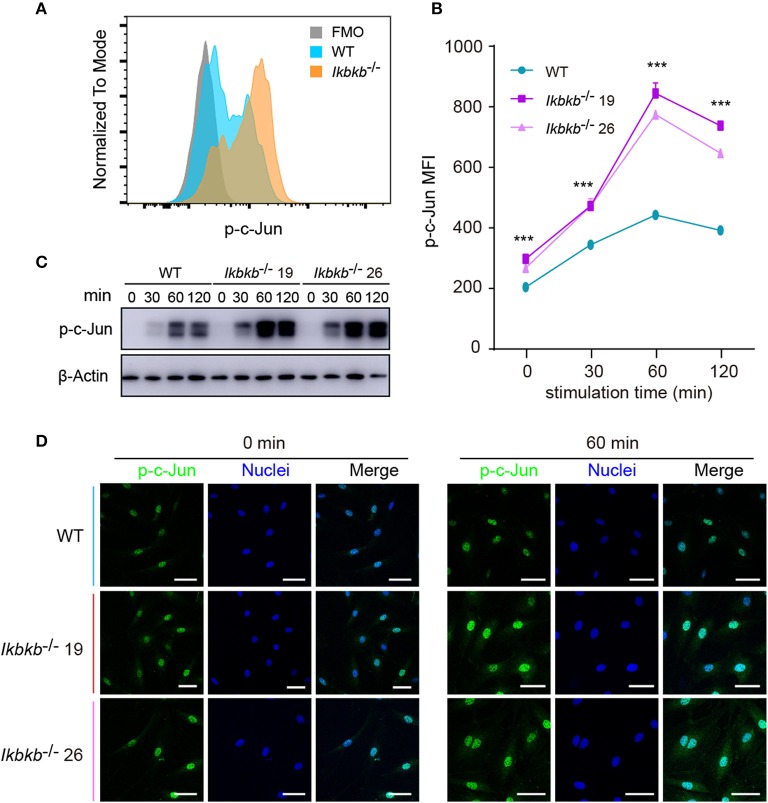
Phosphorylation of c-Jun in wildtype (WT) and IKKβ-deficient MC3T3–E1 cells. **(A,B)** Representative data of phospho-c-Jun **(A)** at 60 min after β-GP and AA stimulation, and mean fluorescence intensity (MFI) of phospho-c-Jun **(B)** at 0, 60, and 120 min after β-GP and AA treatment, compared between WT and IKKβ-deficient cells (*n* = 5). Statistics by one-way ANOVA. **(C)** Western blot analysis for phospho-c-Jun proteins in WT and IKKβ-deficient MC3T3–E1 cells after the treatment described in B. Images were representatives of three independent experiments. **(D)** Representative immunofluorescence images of phospho-c-Jun in WT and IKKβ-deficient MC3T3–E1 cells after 0, 60 min treatment with β-GP and AA. Scale bars: 50 μm. Error bars represent ± SEM. ***P < 0.001, ns = not significant.

JNK is a critical kinase for cell differentiation and triggers c-Jun activation via phosphorylation the N-terminal domain of the protein at Ser63 and Ser73 (12), which synergizes with other transcription factors to influence activator protein-1 (AP-1) activity (26). While several AP-1 subunits have been proved to be important for bone formation (27), c-Jun itself for the embryonic lethality remains incompletely understood for its function in osteoblasts. We first quantitatively analyzed c-Jun phosphorylation of IKKβ-deficient MC3T3-E1 cells by flow cytometry after intracellular staining of anti- phosphorylated form of c-Jun (Ser63). Significant increase of c-Jun phosphorylation was found in IKKβ knockout cells after osteogenic medium treatment at different time points (Figures 4A,B). In line with the single cell analysis by FACS, the Western blot experiments detected obviously increased phosphorylation of c-Jun, with maximal band intensity found following 60 min treatment of osteogenic medium in the same two independent clones (Figure 4C). Under confocal microscope phosphorylated c-Jun was found to be stringently localized in the nucleus (Figure 4D). The experiments above provided evidence that IKKβ-deficient MC3T3-E1 cells had increased JNK phosphorylation and hyperactive c-Jun activity associated with dramatically increased osteogenesis.

### Genetic Inactivation of JNK1 or JNK2 Completely Abolishes IKKβ Deficiency-Sensitized Osteoblast Differentiation and Mineralization

**Figure 5:**
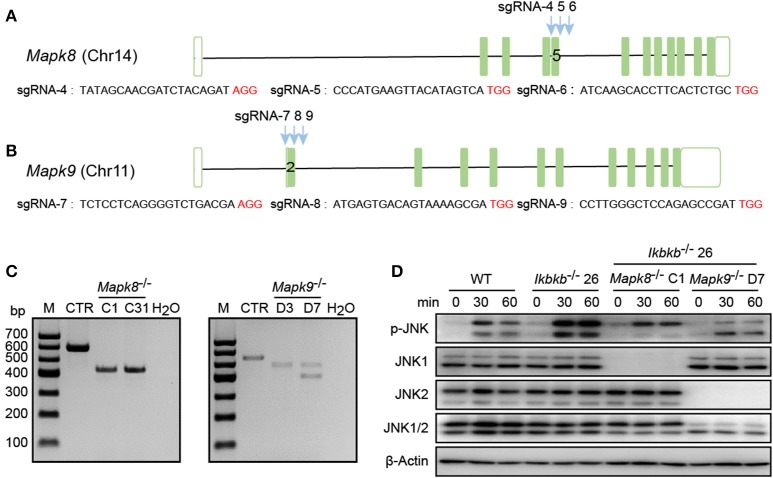
Deletion of JNK1 or JNK2 in IKKβ-deficient MC3T3–E1 cells. **(A,B)** Schematic diagram showing the adjacently placed sgRNAs designed to target mouse *Mapk8* or *Mapk9*, which are located on different murine chromosomes. PAM motifs were shown in red color. **(C)** Genomic edition results of representative clones of *Mapk8*^−/−^ or *Mapk9*^−/−^ validated by PCR and agarose electrophoresis in *Ikbkb*^−/−^ clone 26. Control (CTR) referred to *Mapk8* or *Mapk9* untargeted *Ikbkb*^−/−^ clone 26 and H_2_O was used as negative control. **(D)** MC3T3–E1 wildtype (WT), *Ikbkb*^−/−^ clone 26, *Ikbkb*^−/−^ *Mapk8*^−/−^ clone C1, and *Ikbkb*^−/−^ *Mapk9*^−/−^ clone D7 were treated with β-GP and AA for indicated time points. Western blot analysis was performed with the indicated antibodies. Images were representatives of three independent experiments.

In our study, significant increase in JNK phosphorylation was found to be concomitant with hyperactive c-Jun during osteogenic differentiation in the absence of IKKβ. Since transcripts derived from the two different genes *Mapk8* and *Mapk9* encode proteins with and without a COOH-terminal extension to create both 46 and 54 kDa isoforms (12), and both of the two isoforms were observed significantly increase in phosphorylation in our study, we performed further experiments using CIRSPR/Cas9 genome editing tool in IKKβ-deficient MC3T3-E1 cells to elucidate functional basis of JNK1 and JNK2 in IKKβ deficiency-sensitized osteoblast differentiation. It is important to note that JNK1 and JNK2 are encoded by two different genes localized on different murine chromosomes. To inactivate JNK1 and JNK2 in IKKβ-deficient MC3T3-E1 cells, we selected a representative clone of IKKβ knockout cells which was further subjected to transfection and FACS assay of JNK1 or JNK2 targeted cells as described above. *Mapk8* the gene encoding JNK1 was targeted by three sgRNAs at exon 5 and *Mapk9* encoding JNK2 was targeted by three sgRNAs at exon 2. Chromosomal localization of *Mapk8* and *Mapk9* are on Chr14 and Chr11, respectively (Figures 5A,B). For both *Mapk8* and *Mapk9* knockout experiments, we isolated two independent clones for *Ikbkb*^−/−^ *Mapk8*^−/−^ or *Ikbkb*^−/−^ *Mapk9*^−/−^ double knockout cells as detected by PCR and agarose gel electrophoresis. Two independent clones of *Ikbkb*^−/−^ *Mapk8*^−/−^ clones were designated as C1, C31, *Ikbkb*^−/−^ *Mapk9*^−/−^ clones were designated as D3, D7 (Figure 5C). *Ikbkb*^−/−^ *Mapk8*^−/−^ or *Ikbkb*^−/−^ *Mapk9*^−/−^ double knockout cells were sequenced and validated large DNA fragment deletion in the targeted exons (Supplementary Figure 4A). We analyzed phosphorylation of JNK in *Ikbkb*^−/−^ *Mapk8*^−/−^ or *Ikbkb*^−/−^ *Mapk9*^−/−^ double knockout cells after stimulated with β-GP and AA for indicated time points, by comparing to that of *Ikbkb*^−/−^ cells. We found that JNK1 and JNK2 are both phosphorylated during induced osteoblast differentiation, and Western blot detected complete absence of JNK1 or JNK2 protein in *Mapk8*^−/−^ or *Mapk9*^−/−^ cells (Figure 5D).

**Figure 6:**
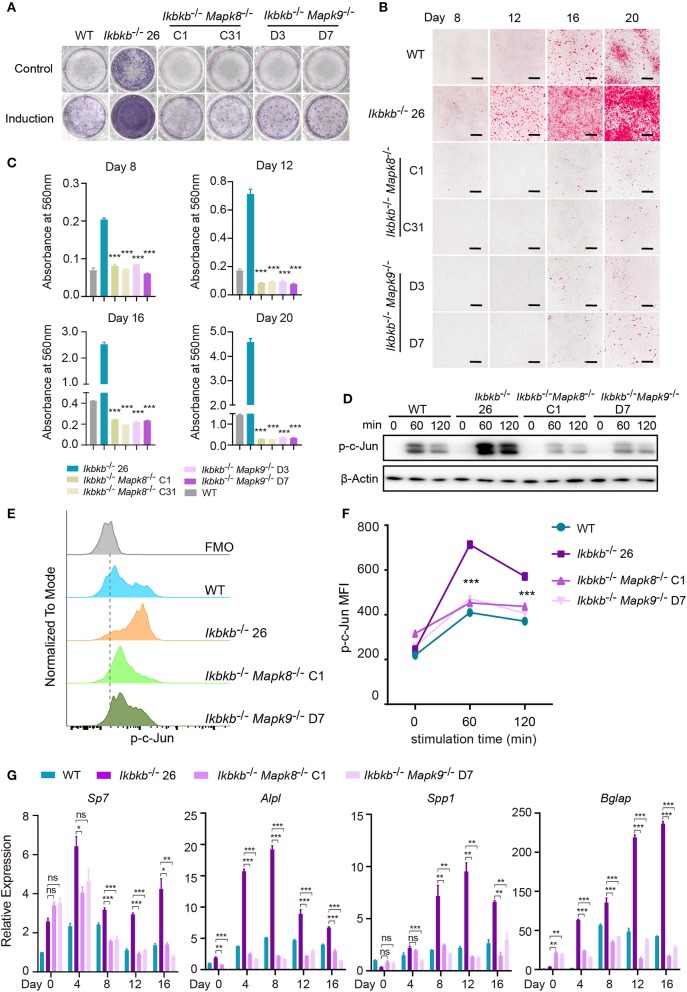
JNK1 or JNK2 deficiency can both decrease IKKβ deficiency-sensitized osteoblast differentiation and mineralization. **(A)** ALP staining in *Ikbkb*^−/−^, *Ikbkb*^−/−^ *Mapk8*^−/−^, and *Ikbkb*^−/−^ *Mapk9*^−/−^ cells after 8 days of control medium or osteogenic medium incubation. **(B)** Microscopy images of Alizarin Red S staining of *Ikbkb*^−/−^, *Ikbkb*^−/−^ *Mapk8*^−/−^, and *Ikbkb*^−/−^ *Mapk9*^−/−^ cells induced by osteogenic medium for indicated days. **(A,B)** Results were representatives of three independent experiments. **(C)** Decreased mineralization in JNK1 or JNK2 deficiency cells as determined by de-staining Alizarin Red S and measuring absorbance at 560 nm (*n* = 3), compared with *Ikbkb*^−/−^ cells. Statistics by one-way ANOVA. **(D)** Western blot analysis for phospho-c-Jun in wildtype (WT), *Ikbkb*^−/−^, *Ikbkb*^−/−^ *Mapk8*^−/−^, and *Ikbkb*^−/−^ *Mapk9*^−/−^ cells after stimulated with β-GP and AA for indicated times points. **(E,F)** WT, *Ikbkb*^−/−^ knock out, *Ikbkb*^−/−^ *Mapk8*^−/−^, and *Ikbkb*^−/−^ *Mapk9*^−/−^ double knock out cells were stimulated with β-GP and AA, and phosphorylation of c-Jun was analyzed by flow cytometry. **(E)** Histogram comparing c-Jun phosphorylation of WT, *Ikbkb*^−/−^, *Ikbkb*^−/−^ *Mapk8*^−/−^, and *Ikbkb*^−/−^ *Mapk9*^−/−^ cells 60 min after stimulation. **(F)** Mean fluorescence intensity (MFI) of phospho-c-Jun at 0, 60, and 120 min with β-GP and AA treatment (*n* = 3). Statistics by one-way ANOVA. **(G)** Real-time PCR results of mRNA levels of osteoblast differentiation marker genes, which showed significant decrease in JNK1 or JNK2 deficient cells (*n* = 3), compared with *Ikbkb*^−/−^ cells. Statistics by one-way ANOVA. Scale bars: 500 μm. Error bars represent ± SEM. *P < 0.05, **P < 0.01, ***P < 0.001, ns = not significant.

Using the *Ikbkb*^−/−^ *Mapk8*^−/−^ or *Ikbkb*^−/−^ *Mapk9*^−/−^ double knockout cells we performed functional analyses to determine contribution of JNK1 and JNK2 to IKKβ deficiency-sensitized osteoblast differentiation and matrix mineralization. *Ikbkb*^−/−^ *Mapk8*^−/−^ or *Ikbkb*^−/−^ *Mapk9*^−/−^ double knockout cells were cultured with osteogenic medium for indicated days, in contrast to *Ikbkb*^−/−^ cells, both cells showed significantly decreased ALP activity (Figure 6A). Also, deletion of JNK1 or JNK2 led to obviously lower level of matrix mineralization in *Ikbkb*^−/−^cells as measured by Alizarin Red S staining (Figures 6B,C; Images of 6-well plates presented in Supplementary Figure 4B). Interestingly, we found that c-Jun phosphorylation measured by Western blot was dramatically inhibited in *Ikbkb*^−/−^ *Mapk8*^−/−^ or *Ikbkb*^−/−^ *Mapk9*^−/−^ double knockout cells in comparison to *Ikbkb*^−/−^ cells treated with β-GP and AA (Figure 6D). As described above, we further used flow cytometry to analyze c-Jun phosphorylation in *Ikbkb*^−/−^ *Mapk8*^−/−^ or *Ikbkb*^−/−^ *Mapk9*^−/−^ double knockout cells with *Ikbkb*^−/−^ cells as controls. At two different time points, 60 and 120 min of β-GP and AA treatment potently induced c-Jun phosphorylation in *Ikbkb*^−/−^ cells which was 1.7 and 1.5-fold higher than the *Ikbkb*^+/+^ WT cells under the same treatments. However, such induced c-Jun phosphorylation was apparently decreased by JNK1 or JNK2 deficiency (Figures 6E,F). As shown in Figure 6G, osteoblast differentiation markers including *Sp7, Alpl, Spp1, Bglap* which had increased in *Ikbkb*^−/−^ cells following induction were drastically decreased in the absence of either JNK1 or JNK2 in *Ikbkb*^−/−^ *Mapk8*^−/−^ or *Ikbkb*^−/−^ *Mapk9*^−/−^ double knockout cells when analyzed at day 4, 8, 12, 16 of osteogenic induction. In further analyses, we compared WT cell expression of differentiation markers in control medium or induction medium, and the same comparison was performed for IKKβ-deficient cells, as well as *Ikbkb*^−/−^ *Mapk8*^−/−^ or *Ikbkb*^−/−^ *Mapk9*^−/−^ double knockout cells. It was obvious that at all time points, marker genes expression induced by osteogenic medium were inhibited by JNK1 or JNK2 deficiency (Supplementary Figure 4C). Our data from genetic models involving *Ikbkb*^−/−^ *Mapk8*^−/−^ or *Ikbkb*^−/−^ *Mapk9*^−/−^ double knockout cells showed clearly that both JNK1 and JNK2 are important for c-Jun activation and that IKKβ deficiency-sensitized osteogenesis could be completely abolished in the absence of either JNK1 or JNK2.

### Transcriptomic Response to Osteogenic Induction in *Ikbkb*^−/−^ Cells Is Maintained by Both JNK1 and JNK2

**Figure 7:**
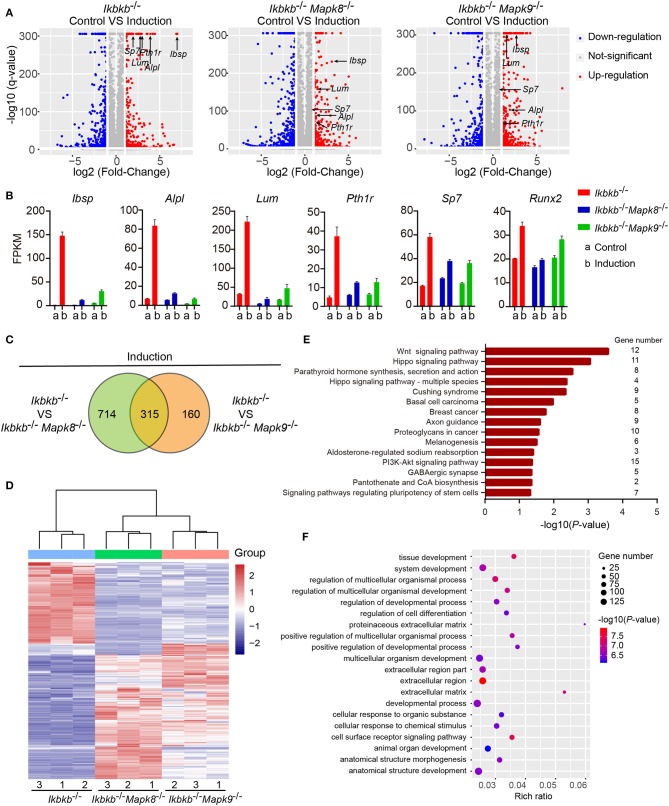
Influence of JNK1 or JNK2 deficiency on *Ikbkb*^−/−^ cells at transcriptomics level. **(A)** Volcano plot shows mRNAs of DEGs between control groups and osteogenic induction groups involving three types of cells namely *Ikbkb*^−/−^, *Ikbkb*^−/−^ *Mapk8*^−/−^, and *Ikbkb*^−/−^ *Mapk9*^−/−^ cells. The horizontal axis represents the fold changes between control groups and osteogenic induction groups. The vertical axis represents the *q*-value of the difference. Representative DEGs were pointed out by arrows to illustrate the gene expression changed by JNK1 or JNK2 deficiency during the response of IKKβ-deficient cells to osteogenic induction at day 4. **(B)** FPKMs of mRNAs of representative DEGs analyzed by RNA-seq. **(C)** DEGs of *Ikbkb*^−/−^ *Mapk8*^−/−^ cells compared with *Ikbkb*^−/−^ cells and DEGs of *Ikbkb*^−/−^ *Mapk9*^−/−^ cells compared with *Ikbkb*^−/−^ cells after 4 days of induction were classified into two categories in Venn diagram, the overlapping DEGs could be regulated by both JNK1 and JNK2. **(D)** Clustering results of the overlapping DEGs described in C. Expression profiles of these overlapping DEGs in *Ikbkb*^−/−^ *Mapk8*^−/−^ cells and *Ikbkb*^−/−^ *Mapk9*^−/−^ cells after induction were very similar in pattern. **(E,F)** KEGG pathway analysis and GO analysis of overlapping DEGs described in C. **(E)** The top 15 enrichment scores (–log10, *P*-value) of KEGG pathways, term candidate gene numbers were listed on the right. **(F)** GO analysis with top 20 representative most significantly enriched (–log10, *P*-value) GO terms of the overlapping DEGs covering biological processes and cellular components domains. Error bars represent ± SEM.

To decipher in an un-biased manner the underlying mechanisms for the fact that JNK1 and JNK2 are indispensably required during IKKβ deficiency-sensitized osteogenesis, we performed whole genome mRNA sequencing involving *Ikbkb*^−/−^ *Mapk8*^−/−^ or *Ikbkb*^−/−^ *Mapk9*^−/−^ double knockout MC3T3-E1 cells and *Ikbkb*^−/−^ cells, under steady state and osteogenic induction conditions. Cells were cultured in basic medium or subjected to osteogenic medium induction for 4 days before extraction of RNA. We compared at transcriptomic level the alteration of gene expression in *Ikbkb*^−/−^ *Mapk8*^−/−^ or *Ikbkb*^−/−^ *Mapk9*^−/−^ double knockout MC3T3-E1 cells following osteogenic medium treatment in comparison to *Ikbkb*^−/−^ cells. We selected representative genes with the highest fold change (top 20), such genes included *Ibsp, Alpl, Lum, Pth1r*, and another 2 osteogenic markers genes. *Lum* and *Pth1r* have relevance to osteogenesis as reported in previous work (28, 29). As expected, the representative genes related to osteoblast differentiation highly upregulated in *Ikbkb*^−/−^ cells following induction with osteogenic medium were not as notably upregulated in *Ikbkb*^−/−^ *Mapk*8^−/−^ or *Ikbkb*^−/−^ *Mapk*9^−/−^ double knockout cells (Figure 7A). As shown in Figure 7B, FPKMs of mRNA quantity for the representative genes were significantly changed by osteogenic induction in *Ikbkb*^−/−^ cells. Strikingly, *Ikbkb*^−/−^ *Mapk*8^−/−^ or *Ikbkb*^−/−^ *Mapk*9^−/−^ double knockout cells displayed significantly less dramatic changes in FPKM of these mRNAs following osteogenic medium treatment.

In further analyses, we aimed to figure out the rationale at transcriptomic level for the observation that both JNK1 and JNK2 were required for potentiated osteoblast differentiation on IKKβ-deficient background. As shown in Figure 7C, we noted that, following induction medium treatment, 66.3% of the genes (315 out of 475) altered by JNK2 deficiency (by comparing *Ikbkb*^−/−^ *Mapk*9^−/−^ double knockout cells to *Ikbkb*^−/−^ cells treated with osteogenic medium) were shared by those caused by JNK1 deficiency (by comparing *Ikbkb*^−/−^ *Mapk*8^−/−^ double knockout cells to *Ikbkb*^−/−^ cells treated with osteogenic medium). Furthermore, we performed hierarchical clustering analysis of such 315 genes. Interestingly, these genes were expressed in similar pattern in *Ikbkb*^−/−^ *Mapk*8^−/−^ or *Ikbkb*^−/−^ *Mapk*9^−/−^ cells, while they were conversely correlated to the expression profile in *Ikbkb*^−/−^ cells (Figure 7D). The result indicated that both JNK1 and JNK2 could play similar roles in transcriptional regulation during IKKβ deficiency-promoted osteoblast differentiation. Since both JNK1 and JNK2 were found to be essential for enhanced differentiation and mineralization in IKKβ-deficient cells, and we performed Gene Ontology (GO) and Kyoto Encyclopedia of Genes and Genomes (KEGG) pathway analyses to identify biological processes and pathways requiring both JNK1 and JNK2 to regulate the 315 overlapping differentially expressed genes (DEGs). GO analysis revealed that these 315 DEGs were involved in biological processes as cell surface receptor signaling pathway, tissue development, cellular response to chemical stimulus. We found that pathways including Wnt signaling pathway, PI3K-Akt signaling pathway were enriched in the 315 genes, suggesting involvement of such pathways in IKKβ deficiency-sensitized osteogenesis (Figures 7E,F). More importantly, our data at transcriptomic level proved that JNK1 and JNK2 function similarly in promoting differentiation and mineralization in IKKβ-deficient preosteoblast cells.

## Discussion

Although NF-κB is crucial for transcriptional regulation of broad range of biological processes (30), its roles in osteoblast differentiation and extracellular matrix mineralization remain not completely elucidated, and some of the previous studies generated inconsistent results. Previous studies using NF-κB inhibitors and RNA interference showed that NF-κB inhibition impaired osteoblastic-like differentiation and matrix mineralization in vascular smooth muscle cells (VSMC) (31, 32). However, A more recent study of vascular calcification using IKKβ-deficient murine model showed that genetic deletion of a critical member of the NF-κB activator IKK complex IKKβ resulted in increased matrix mineralization in VSMC extracted from IKKβ knockout mice (33), which was completely contradictory to the study using IKKβ inhibitors (31). On the other hand, in osteoblast cells inhibition of NF-κB was found to increase osteoblast differentiation and mineralization (9). Above such controversies, the underlying mechanisms how NF-κB deficiency influence matrix mineralization remain poorly understood.

In this study, we applied CRISPR/Cas9 genome editing tools to establish IKKβ knockout cells by deleting *Ikbkb* gene both in primary osteoblast cells and preosteoblast cells MC3T3-E1. In MC3T3-E1 cells, multiple individual clones completely deprived of IKKβ protein and bulk-sorted IKKβ-deficient cells determined that IKKβ deficiency could potently sensitize mineralization induced by osteogenic medium. We treated the cells with osteogenic medium (10 mM β-GP and 50 μg/ml AA) as reported in previous studies (34, 35) for osteoblast differentiation and mineralization. We observed obvious matrix mineralization in primary osteoblasts of B6 mice in such induction condition even though in our experiments the MC3T3–E1 WT cells had much lower level of ALP activity and matrix mineralization in comparison to that of the IKKβ-deficient cells, and the Alizarin Red staining of matrix mineralization in WT cells was visible from day 16, and more apparent at day 20 during osteogenic induction. When we cultured the MC3T3–E1 WT cells for 4 weeks instead of 3 weeks in the same induction medium, the matrix mineralization in WT cells was much more obvious, which was accompanied by significant increase of osteoblast differentiation markers expression (Supplementary Figures 5A,B). In addition, we examined promotive effects of IKKβ-deficiency on matrix mineralization involving the bone morphogenetic protein BMP-2 in *Ikbkb* knockout cells with WT MC3T3–E1 cells as controls (36), and we found that in the presence of BMP-2, IKKβ-deficient MC3T3–E1 cells had obviously stronger mineralization (Supplementary Figure 6). Such genetic models could provide highly efficient system to perform functional and mechanistic studies for osteoblast differentiation and mineralization. Using the IKKβ deficiency model, we first analyzed JNK and c-Jun axis for their roles in increased osteogenesis since JNK phosphorylation can be readily induced by β-GP and AA treatment in IKKβ deficiency cells and our IKKβ-deficient model exhibited exaggerated response to such treatments. In the control medium, the phosphorylation levels of JNK and c-Jun in IKKβ-deficient osteoblasts were also higher than those in WT cells (Supplementary Figure 7). In additional experiments, we stimulated the IKKβ-depleted MC3T3-E1 cells and WT control cells with BMP-2, and found that treatment with BMP-2 was sufficient to trigger JNK phosphorylation in mutant and WT cells. However, the BMP-2 induced JNK phosphorylation was significantly enhanced in IKKβ-depleted cells (Supplementary Figure 8). Although previous studies proved that inhibition of IKK/NF-κB could activate JNK (9, 37, 38), the functional validation in osteogenesis was not performed. More importantly JNK1 and JNK2 encoded by two different genes *Mapk8* and *Mapk9*, and their specific role in mineralization especially under the background of IKKβ-deficient has not been elucidated. As IKKβ deficiency highly sensitizes osteoblast differentiation following osteogenic induction medium treatment, we therefore further deleted JNK1 or JNK2, respectively, in the same *Ikbkb*^−/−^ clone to generate *Ikbkb*^−/−^ *Mapk*8^−/−^ or *Ikbkb*^−/−^ *Mapk*9^−/−^ double knockout cells to determine specific roles of JNK1 and JNK2 during osteoblast differentiation and mineralization. It is important to note that previous study was not likely to differentiate JNK1 and JNK2 function in matrix mineralization sensitized by IKK/NF-κB inhibition (9). Our double knockout model involving JNK1 or JNK2 explicitly determined that phosphorylation of both JNK1 and JNK2 occurred. In further functional studies, we found that loss of either JNK1 or JNK2 was sufficient to abolish completely sensitized matrix mineralization in IKKβ-deficient cells. It is also important to note that IKKβ-deficient osteoblasts had background ALP activity in control medium which was absent in *Ikbkb*^−/−^ *Mapk8*^−/−^ and *Ikbkb*^−/−^ *Mapk9*^−/−^ cells, suggesting that both JNK1 and JNK2 are required for elevated basal level of ALP activity in IKKβ-deficient osteoblasts. Such results confirmed that both JNK1 and JNK2 are activated and functionally required during osteobalst differentiation and matrix mineralization. As we observed extremely strong phenotype of matrix mineralization in IKKβ-deficient osteoblasts, and elucidated the crucial function of both JNK1 and JNK2 for the sensitized phenotype, it remains interesting to investigate whether these two kinases function similarly on the WT background in future studies.

Since *Ikbkb*^−/−^ *Mapk8*^−/−^ and *Ikbkb*^−/−^ *Mapk*9^−/−^ double knockout cells displayed identical phenotype, we compared c-Jun transcriptional activity by measuring its phosphorylation and abundance in nucleus. Surprisingly, both JNK1 and JNK2 were found required for the phosphorylation of c-Jun. Therefore, by using the genetic models, we discovered that JNK1 and JNK2 are both phosphorylated during induced osteogenic differentiation, and more importantly, they are both required for c-Jun phosphorylation. Even though previous studies (9, 14) showed that JNK phosphorylation was induced by β-GP and AA, it was not determined that both JNK1 and JNK2 are phosphorylated and they are both essential for c-Jun activation. Since the other factors modulated by JNK such as ATF4 and Smad1 are also contributing to osteogenic differentiation (14, 39, 40), we also examined the expression of ATF4 and the activation of Smad1 in both IKKβ-deficient MC3T3–E1 cells and WT control cells. We found that the hyperactivity of JNK in IKKβ-deficient MC3T3–E1 cells did not result in higher level of ATF4 expression. When analyzing the phosphorylation of Smad1, we also did not found detectable differences by Western blot between IKKβ-deficient MC3T3–E1 cells and WT controls following 30 and 60 min of osteogenic medium stimulation (Supplementary Figures 2D,E).

In addition, we tried to know at transcriptomic level, which genes were commonly regulated by JNK1 and JNK2, when IKKβ-deficient cells were treated with induction medium. Therefore, we compared IKKβ-deficient cells with *Ikbkb*^−/−^ *Mapk8*^−/−^ or *Ikbkb*^−/−^ *Mapk9*^−/−^ double knockout cells for their transcriptomic responses of osteogenic induction. It was surprising to find that 315 genes out 475 genes modulated by JNK2 deficiency (by comparing *Ikbkb*^−/−^ *Mapk9*^−/−^ double knockout cells to *Ikbkb*^−/−^ cells treated with osteogenic medium) were included in the genes modulated by JNK1 deficiency (by comparing *Ikbkb*^−/−^ *Mapk8*^−/−^ double knockout cells to Ikbkb^−/−^ cells treated with osteogenic medium). We isolated the genes that were induced in *Ikbkb*^−/−^ knockout cells during mineralization, and surprisingly we found that such gene expression patterns in heat map clusters were reversed quite similarly by further JNK1 or JNK2 knockout. Such unbiased analyses shed light on the conserved function of JNK1 and JNK2 in transducing osteogenic signals in IKKβ-deficient cells. KEGG pathway enrichment analysis of these differentially expressed 315 genes which were regulated by both JNK1 and JNK2 revealed that Wnt signaling pathway and PI3K-Akt signaling pathway could be implicated in blockade of osteogenesis sensitized by IKKβ deficiency. Since Wnt signaling pathway was found as a central pathway in osteogenesis (41–43), our unbiased genomic analyses shed light on how JNK1 and JNK2 activation could trigger synergistically gene expression involving in osteogenesis by using three type of genetically engineered cellular models, *Ikbkb*^−/−^ knockout cells, *Ikbkb*^−/−^ *Mapk8*^−/−^ and *Ikbkb*^−/−^ *Mapk9*^−/−^ double knockout cells. However, more functional validation of the gene clusters we identified from such genetic models are necessary to obtain more complete understanding how JNK1 and JNK2 exert osteogenic functions indispensably.

In conclusion, our study validated that phosphorylation of JNK1 and JNK2 upregulated, respectively, in IKKβ-deficient and osteogenic induction conditions. Inhibition of JNK1 or JNK2 could prevent matrix mineralization in an extremely efficient manner even in the IKKβ deficiency-sensitized cells. These observations warrant further study of JNK signaling pathway inhibition as a therapeutic strategy to treat disordered matrix mineralization.

## Data Availability Statement

The RNA-seq data for this study can be found in the NCBI Sequence Read Archive (SRA) database (www.ncbi.nlm.nih.gov/sra, accession number SRP219248).

## Ethics Statement

The animal study was reviewed and approved by the committee on animal care at Xinxiang Medical University.

## Author Contributions

LZu, LZh, and QH: study design. QH, ZL, and LZh: experiments conduct. QH, ZL, and LL: data collection and data analysis. QH, LZu, and LZh: drafting manuscript. LZu and LZh: revising manuscript content. All authors: data interpretation and approving final version of manuscript.

### Conflict of Interest

The authors declare that the research was conducted in the absence of any commercial or financial relationships that could be construed as a potential conflict of interest.
