# Supplementary material for: Both JNK1 and JNK2 Are Indispensable for Sensitized Extracellular Matrix Mineralization in IKKβ-Deficient Osteoblasts
Source: Front Endocrinol (Lausanne). 2020 Feb 12;11:13. doi: 10.3389/fendo.2020.00013 (PMC7028708; doi:10.3389/fendo.2020.00013)
Supplement: Supplementary file 1 [file Data_Sheet_1.pdf]

Supplementary Material

Supplementary Table 1. Primers for plasmid construction

sgRNAs	Constructions	Primers for plasmid construction
sgRNA-1	pX458-ECFP-m-*Ikbkb*-KO-1	5’-CACCGGATTTGAGCTGAACCCCGG-3’
		5’-AAACCCGGGGTTCAGCTCAAATCC-3’
sgRNA-2	pX458-ECFP-m-*Ikbkb*-KO-2	5’-CACCGTAGGCTGAACCATCCCAATG-3’
		5’-AAACCATTGGGATGGTTCAGCCTAC-3’
sgRNA-3	pX458-DsRed2-m-*Ikbkb*-KO-3	5’-CACCGCCAGCAGTGGCAAATCATT-3’
		5’-AAACAATGATTTGCCACTGCTGGC-3’
sgRNA-4	pX458-ECFP-m-*Mapk8*-KO-1	5’-CACCGTATAGCAACGATCTACAGAT -3’
		5’-AAACATCTGTAGATCGTTGCTATAC-3’
sgRNA-5	pX458-ECFP-m-*Mapk8*-KO-2	5’-CACCGCCCATGAAGTTACATAGTCA-3’
		5’-AAACTGACTATGTAACTTCATGGGC-3’
sgRNA-6	pX458-DsRed2-m-*Mapk8*-KO-3	5’-CACCGATCAAGCACCTTCACTCTGC-3’
		5’-AAACGCAGAGTGAAGGTGCTTGATC-3’
sgRNA-7	pX458-DsRed2-m-*Mapk9*-KO-1	5’-CACCGTCTCCTCAGGGGTCTGACGA-3’
		5’-AAACTCGTCAGACCCCTGAGGAGAC-3’
sgRNA-8	pX458-DsRed2-m-*Mapk9*-KO-2	5’-CACCGATGAGTGACAGTAAAAGCGA-3’
		5’-AAACTCGCTTTTACTGTCACTCATC-3’
sgRNA-9	pX458-ECFP-m-*Mapk9*-KO-3	5’-CACCGCCTTGGGCTCCAGAGCCGAT-3’
		5’-AAACATCGGCTCTGGAGCCCAAGGC-3’

Supplementary Table 2. Primers used to quantify gene expression in qPCR assay

Gene	Primer sequence
Runx2	Forward: 5’-GCCTCCGCTGTTATGAAAAA-3’
	Reverse: 5’-TGGGGAGGATTTGTGAAGAC-3’
Sp7	Forward: 5’-GGGCTCTCTCTGTTCCCCTA-3’
	Reverse: 5’-GCAAAGGCCTGAGAGGAGTT-3’
Alpl	Forward: 5’-GGGACGAATCTCAGGGTACA-3’
	Reverse: 5’-TTCAAGGTCTCTTGGGCTTG-3’
Bglap	Forward: 5’-AAGCAGGAGGGCAATAAGGT-3’
	Reverse: 5’-CAAGCAGGGTTAAGCTCACA-3’
Spp1	Forward: 5’-TGCACCCAGATCCTATAGCC-3’
	Reverse: 5’-CTTTTCTTCAGAGGACACAGCA-3’
Atf4	Forward: 5’-GAAACCTCATGGGTTCTCCA-3’
	Reverse: 5’-TCTCCAACATCCAATCTGTCC-3’
Gapdh	Forward: 5’-GGTGCTGAGTATGTCGTGGA-3’
	Reverse: 5’-GTGGTTCACACCCATCACAA-3’

Supplementary Figure 1

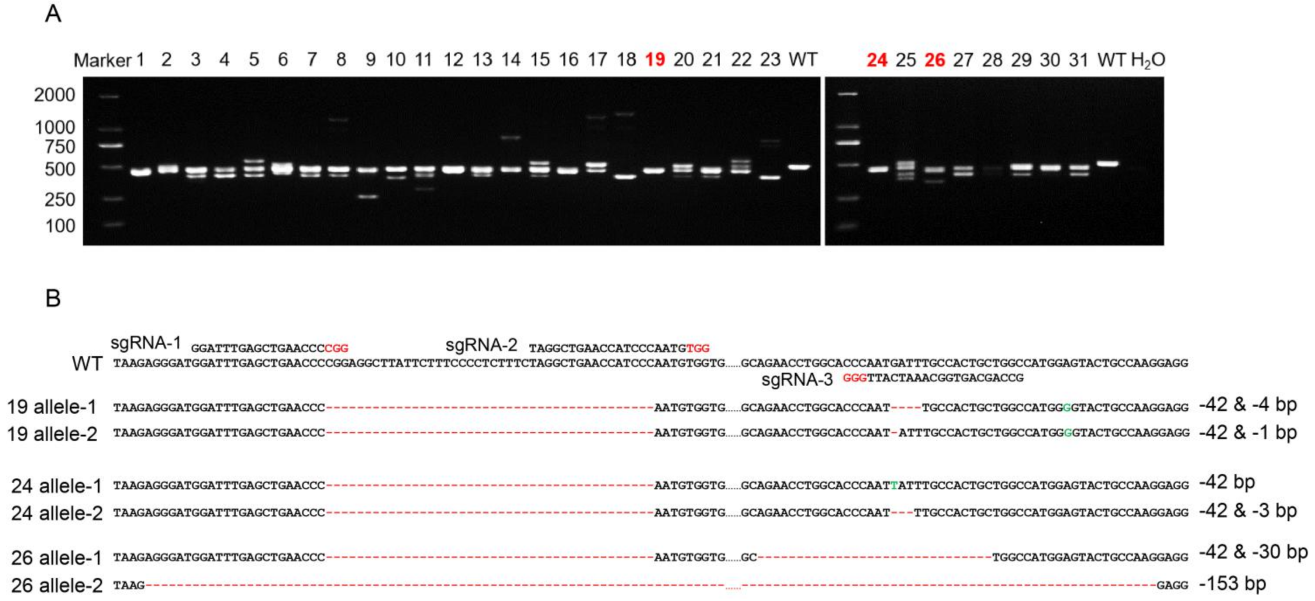

Supplementary Figure 1. Genome editing results of IKKβ-depleted in MC3T3-E1 cells. **(A)** Representative gel image identifying *Ikbkb*$^{-/-}$ clones in preosteoblast cells MC3T3-E1. Three independent mutant clones of MC3T3- E1 cells with deletion of large DNA fragment were obtained by CRISPR/Cas9 genome editing tools and designated as *Ikbkb*$^{-/-}$ clone 19, 24 and 26. H_2O was used as negative control. **(B)** Sanger sequencing data of *Ikbkb*$^{-/-}$ clone 19, 24 and 26 were used to validate genome editing results.

Supplementary Figure 2

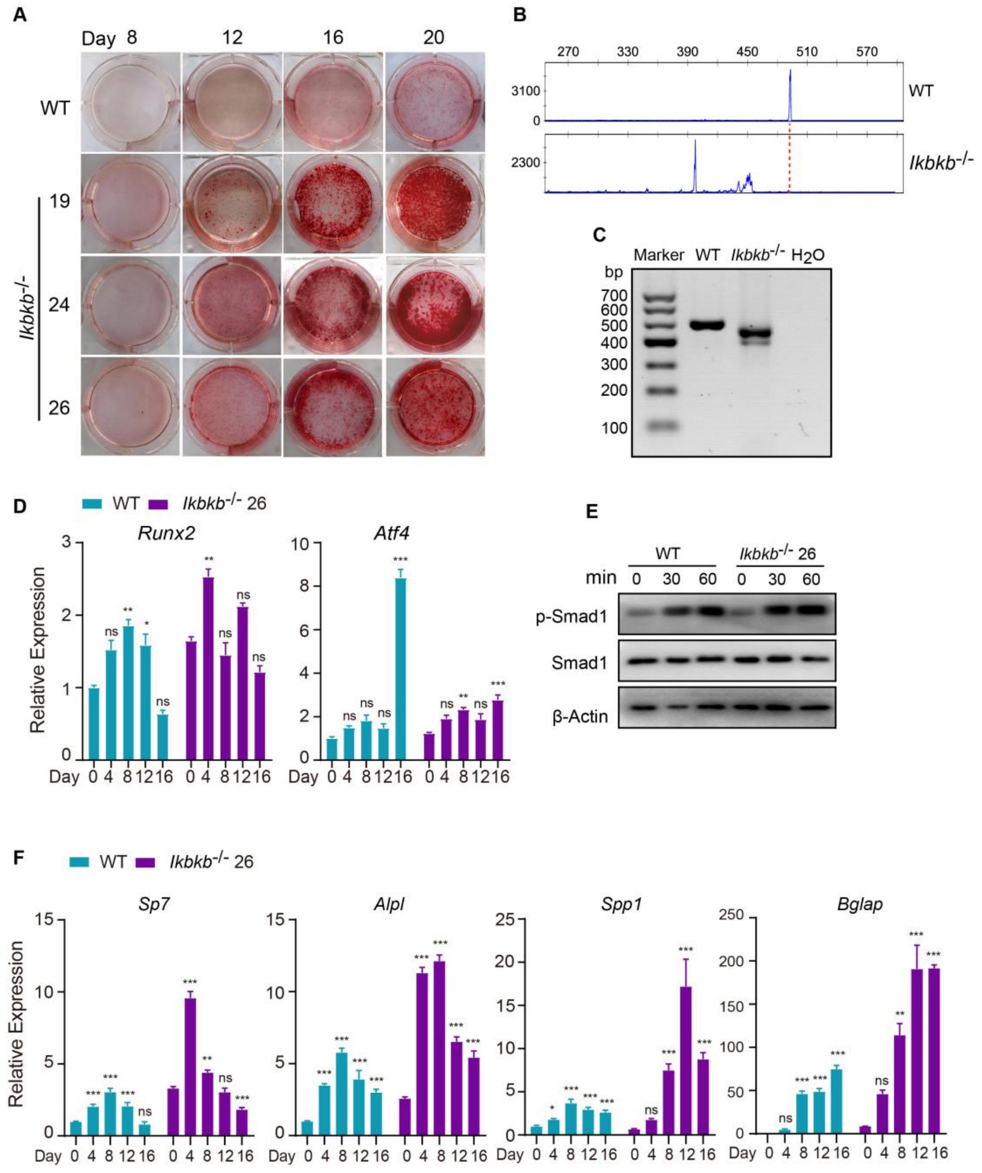

Supplementary Figure 2. Osteogenic differentiation and matrix mineralization in wildtype (WT) and *Ikbkb*$^{-/-}$ cells. **(A)** Alizarin Red S staining of MC3T3-E1 WT control and *Ikbkb*$^{-/-}$ clone 19, 24, 26 induced by osteogenic medium for indicated days. **(B)** A large variety of mutant alleles was identified by capillary array electrophoresis following fluorescent PCR of DNA from expanded 10000 *Ikbkb*$^{-/-}$ bulk-sorted cells. The WT genomic DNA was used as control. **(C)** Genomic edition results of *Ikbkb*$^{-/-}$ bulk-sorted cells validated by PCR and agarose electrophoresis. The WT genomic DNA was used as control. H_2O was used as negative control. **(D)** The mRNA levels of *Runx2* and *Atf4* in *Ikbkb*$^{-/-}$ MC3T3-E1 cells and WT cells determined by real-time PCR (n = 3). Statistics by one-way ANOVA. **(E)** Western blot analysis for phospho-Smad1 in WT and *Ikbkb*$^{-/-}$ MC3T3-E1 cells after stimulating with β-GP and AA for indicated time points. **(F)** Real-time PCR results of mRNA levels of osteoblast differentiation marker genes in WT and *Ikbkb*$^{-/-}$ MC3T3-E1 cells after induced by osteogenic medium for indicated days compared with cells cultured in basic medium (n = 3). Statistics by one-way ANOVA. Error bars represent ± SEM. * $P < 0.05$, ** $P < 0.01$, *** $P < 0.001$, ns = not significant.

Supplementary Figure 3

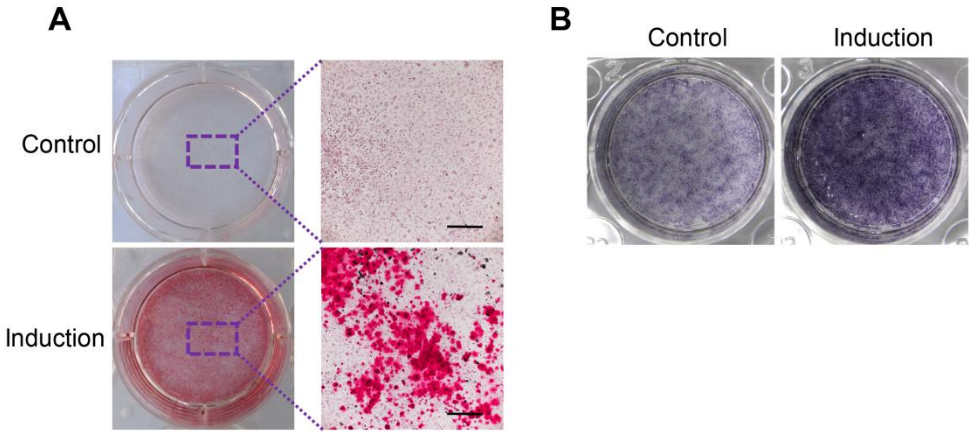

Supplementary Figure 3. Osteogenic induction of primary osteoblasts from neonatal mice. **(A)** Alizarin Red S staining of primary osteoblasts cultured in the osteogenic medium for 21 days. Primary osteoblasts cultured in basic medium were used as control. **(B)** ALP activity of primary osteoblasts was analyzed using ALP staining on day 8 of osteogenic induction. Primary osteoblasts cultured in basic medium were used as control. Scale bars: 500 µm.

Supplementary Figure 4

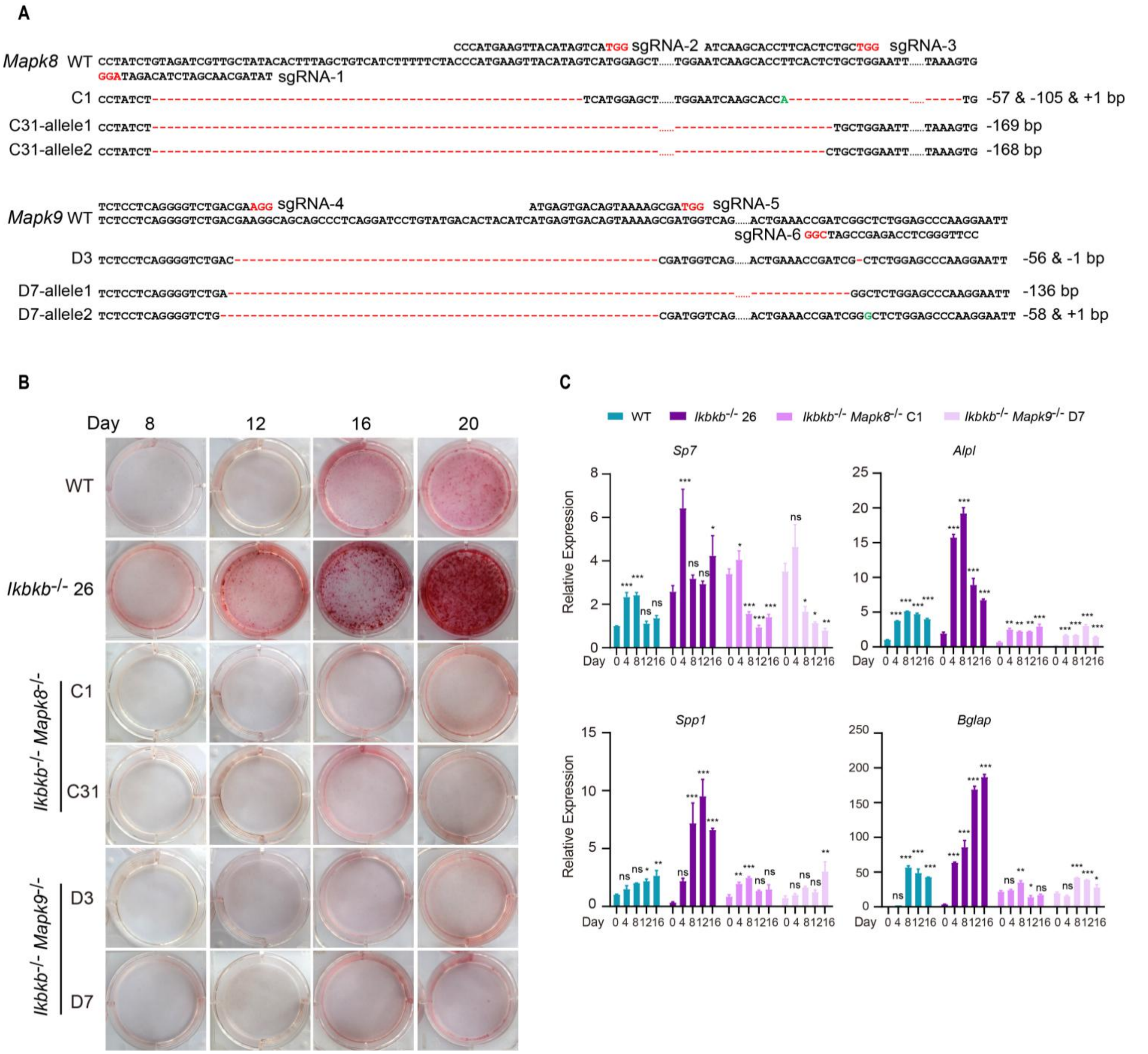

Supplementary Figure 4. (A) Sanger sequencing results indicated different alleles of *Ikbkb*$^{-/-}$ *Mapk8*$^{-/-}$ clones which were designated as C1, C31 by targeting exon 5 of *Mapk8* in *Ikbkb*$^{-/-}$ clone 26. Two independent clones of *Ikbkb*$^{-/-}$ *Mapk9*$^{-/-}$ cells were designated as D3, D7 which were generated by targeting exon 2 of *Mapk9* in *Ikbkb*$^{-/-}$ clone 26. **(B)** Alizarin Red S staining of MC3T3-E1 wildtype (WT), *Ikbkb*$^{-/-}$ clone 26, *Ikbkb*$^{-/-}$ *Mapk8*$^{-/-}$ clone C1, C31 and *Ikbkb*$^{-/-}$ *Mapk9*$^{-/-}$ clone D3, D7 induced by osteogenic medium for indicated days. **(C)** Real-time PCR results of mRNA levels of osteoblast differentiation marker genes in MC3T3-E1 WT, *Ikbkb*$^{-/-}$ clone 26, *Ikbkb*$^{-/-}$ *Mapk8*$^{-/-}$ clone C1, C31 and *Ikbkb*$^{-/-}$ *Mapk9*$^{-/-}$ clone D3, D7 induced by osteogenic medium for indicated days, compared with cells cultured in basic medium (n = 3). Statistics by one-way ANOVA. Error bars represent ± SEM. * $P < 0.05$, ** $P < 0.01$, *** $P < 0.001$, ns = not significant.

Supplementary Figure 5

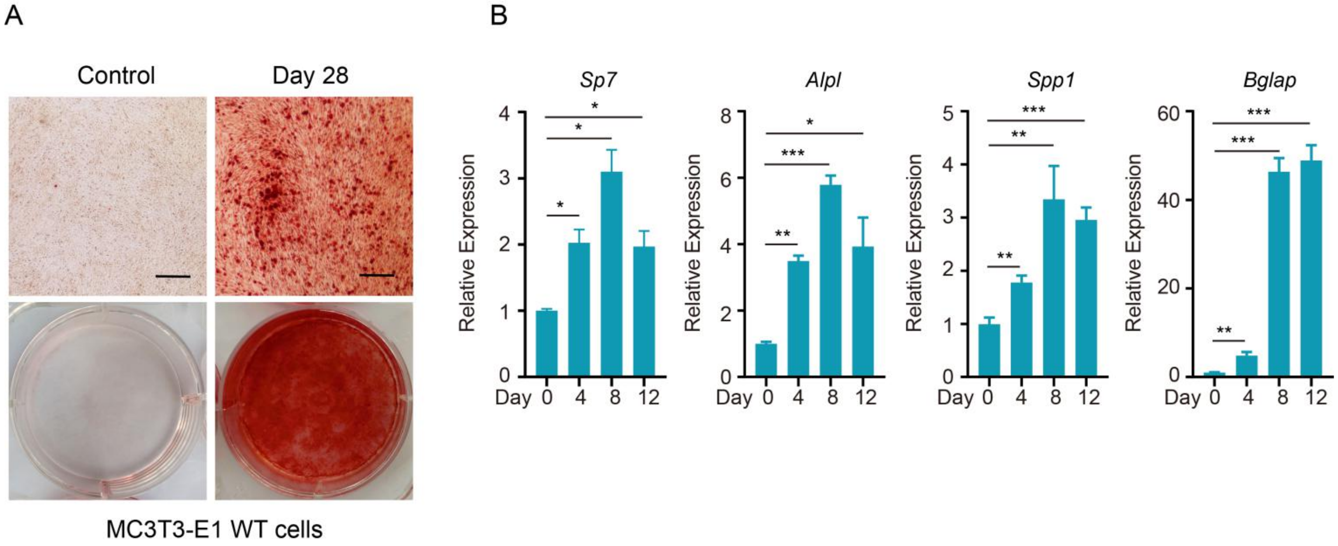

Supplementary Figure 5. MC3T3-E1 wildtype (WT) cells differentiation and mineralization after osteogenic induction. **(A)** Alizarin Red S staining of MC3T3-E1 WT cells cultured in the osteogenic medium for 28 days. WT cells cultured in basic medium were used as control. The results were representatives of three independent experiments. **(B)** The mRNA levels of osteoblast differentiation marker genes determined by real-time PCR (n = 3) in MC3T3-E1 WT cells after induced by osteogenic medium for 4, 8, 12 days compared with day 0. Statistics by two-tailed t-test. Scale bars: 500 µm. Error bars represent ± SEM. * $P < 0.05$, ** $P < 0.01$, *** $P < 0.001$.

Supplementary Figure 6

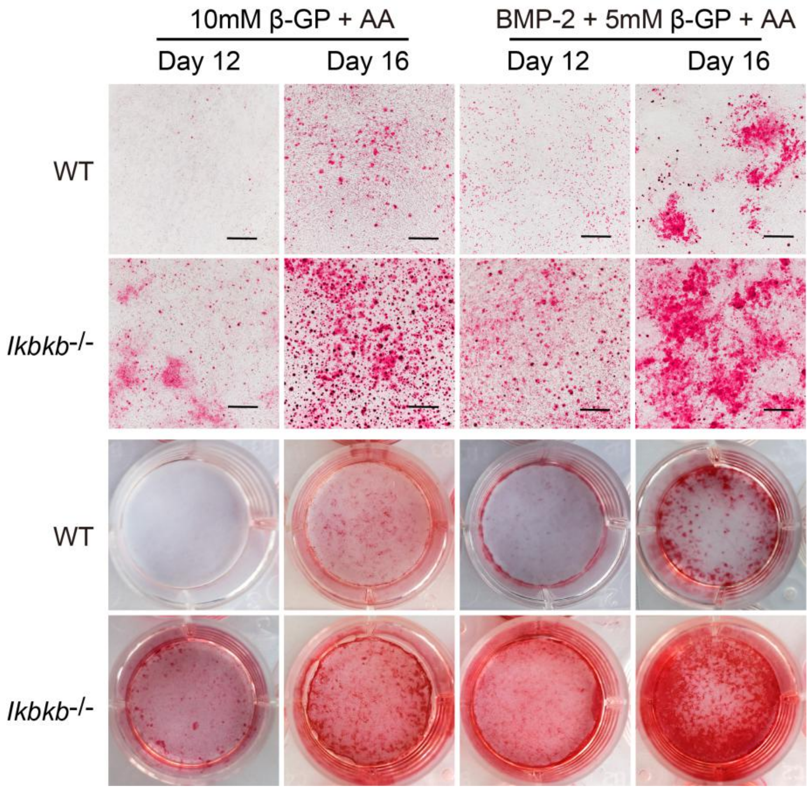

Supplementary Figure 6. Alizarin Red S staining of wildtype (WT) and *Ikbkb*$^{-/-}$ MC3T3-E1 cells induced by osteogenic medium (α-MEM containing 10% FBS, 100 U/ml penicillin, 100 µg/ml streptomycin, 10 mM β-GP and 50 µg/ml AA) and BMP-2 supplemented medium (α-MEM containing 10% FBS, 100 U/ml penicillin, 100 µg/ml streptomycin, 100 ng/ml BMP-2, 5 mM β-GP and 50 µg/ml AA). The results were representatives of three independent experiments. Scale bars: 500 µm.

Supplementary Figure 7

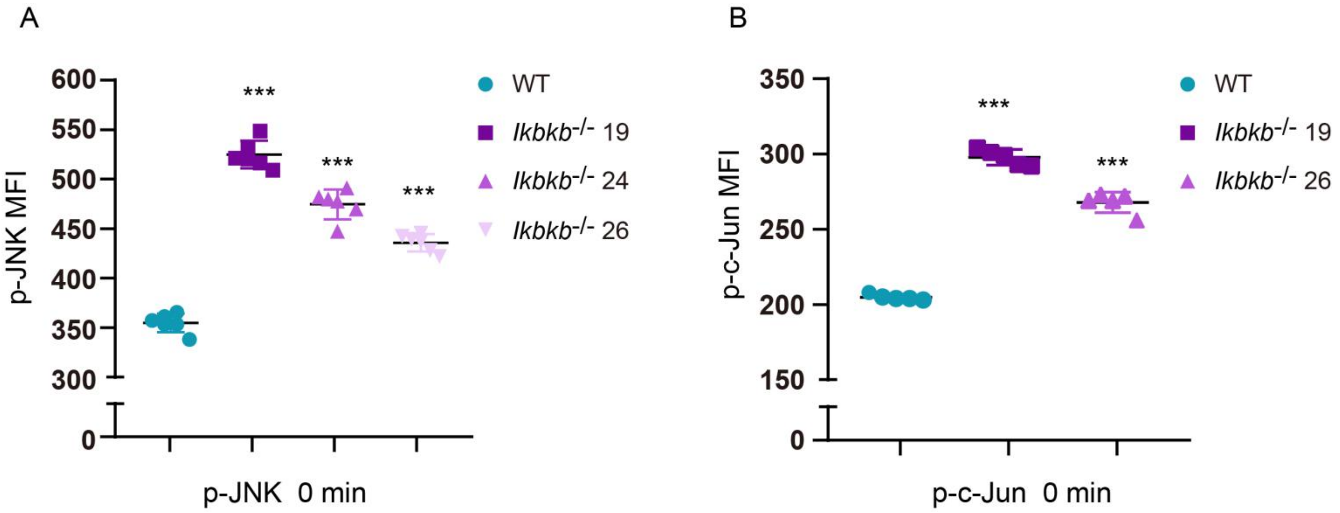

Supplementary Figure 7. The phosphorylation levels of JNK and c-Jun in wildtype (WT) and *Ikbkb*$^{-/-}$ MC3T3-E1 cells in the control medium. **(A)** The phosphorylation levels of JNK in IKKβ-deficient cells in the control medium compared with WT MC3T3-E1 cells (Time 0 data of Figure 3B). Statistics by one-way ANOVA. **(B)** The phosphorylation levels of c-Jun in IKKβ-deficient in the control medium compared with WT MC3T3-E1 cells (Time 0 data of Figure 4B). Statistics by one-way ANOVA. Error bars represent ± SEM. *** $P < 0.001$.

Supplementary Figure 8

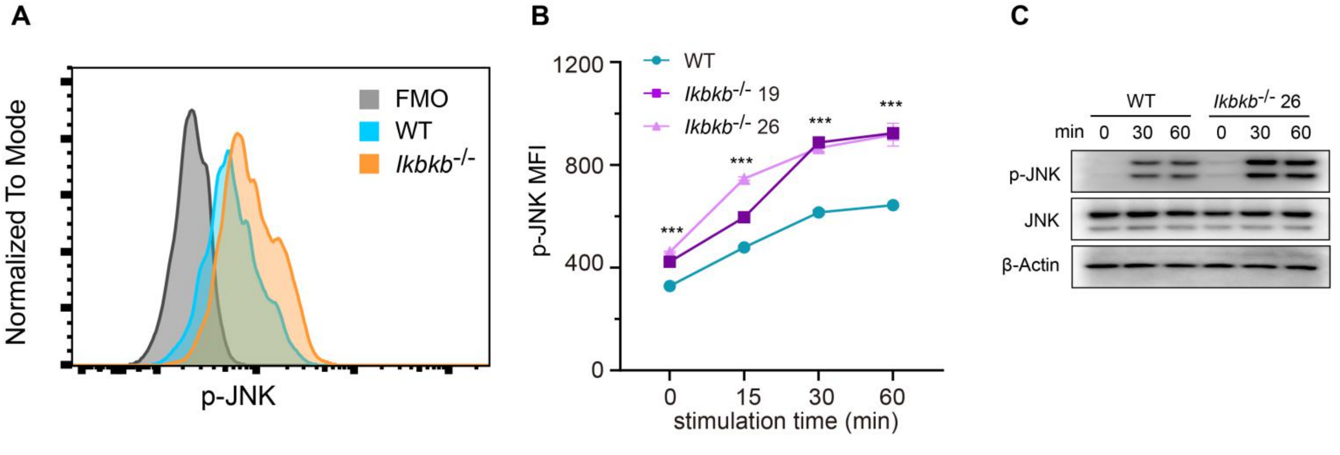

Supplementary Figure 8. Phosphorylation of JNK in wildtype (WT) and *Ikbkb*$^{-/-}$ MC3T3-E1 cells stimulated with BMP-2. **(A)** Histogram comparing mean fluorescence intensity (MFI) of phospho-JNK of WT, *Ikbkb*$^{-/-}$ cells 30 min after BMP-2 stimulation. **(B)** At 0, 15, 30 and 60 min after BMP-2 treatment, comparing between WT and IKKβ-deficient cells (n = 3). Statistics by one-way ANOVA. **(C)** Western blot analysis of phospho-JNK proteins in WT and *Ikbkb*$^{-/-}$ MC3T3-E1 cells after the stimulation with BMP-2. Images were representatives of three independent experiments. Error bars represent ± SEM. *** $P < 0.001$.
